# Development of an ultrasound-based radiomics nomogram to preoperatively predict Ki-67 expression level in patients with breast cancer

**DOI:** 10.3389/fonc.2022.963925

**Published:** 2022-08-15

**Authors:** Jinjin Liu, Xuchao Wang, Mengshang Hu, Yan Zheng, Lin Zhu, Wei Wang, Jisu Hu, Zhiyong Zhou, Yakang Dai, Fenglin Dong

**Affiliations:** ^1^ Department of Ultrasound, The First Affiliated Hospital of Soochow University, Suzhou, China; ^2^ Department of Radiology, The Affiliated Suzhou Hospital of Nanjing Medical University, Suzhou Municipal Hospital, Suzhou, China; ^3^ Suzhou Institute of Biomedical Engineering and Technology, Chinese Academy of Sciences, Suzhou, China

**Keywords:** radiomics nomogram, ultrasonography, breast neoplasms, shear wave elastography (SWE), Ki-67 expression

## Abstract

**Objective:**

To develop and validate a radiomics nomogram that could incorporate clinicopathological characteristics and ultrasound (US)-based radiomics signature to non-invasively predict Ki-67 expression level in patients with breast cancer (BC) preoperatively.

**Methods:**

A total of 328 breast lesions from 324 patients with BC who were pathologically confirmed in our hospital from June 2019 to October 2020 were included, and they were divided into high Ki-67 expression level group and low Ki-67 expression level group. Routine US and shear wave elastography (SWE) were performed for each lesion, and the ipsilateral axillary lymph nodes (ALNs) were scanned for abnormal changes. The datasets were randomly divided into training and validation cohorts with a ratio of 7:3. Correlation analysis and the least absolute shrinkage and selection operator (LASSO) were used to select the radiomics features obtained from gray-scale US images of BC patients, and each radiomics score (Rad-score) was calculated. Afterwards, multivariate logistic regression analysis was used to establish a radiomics nomogram based on the radiomics signature and clinicopathological characteristics. The prediction performance of the nomogram was assessed by the area under the receiver operating characteristic curve (AUC), the calibration curve, and decision curve analysis (DCA) using the results of immunohistochemistry as the gold standard.

**Results:**

The radiomics signature, consisted of eight selected radiomics features, achieved a nearly moderate prediction efficacy with AUC of 0.821 (95% CI:0.764-0.880) and 0.713 (95% CI:0.612-0.814) in the training and validation cohorts, respectively. The radiomics nomogram, incorporating maximum diameter of lesions, stiff rim sign, US-reported ALN status, and radiomics signature showed a promising performance for prediction of Ki-67 expression level, with AUC of 0.904 (95% CI:0.860-0.948) and 0.890 (95% CI:0.817-0.964) in the training and validation cohorts, respectively. The calibration curve and DCA indicated promising consistency and clinical applicability.

**Conclusion:**

The proposed US-based radiomics nomogram could be used to non-invasively predict Ki-67 expression level in BC patients preoperatively, and to assist clinicians in making reliable clinical decisions.

## Introduction

Breast cancer (BC) has surpassed lung cancer to become the most common malignant tumor in the world, with the highest morbidity and mortality among women, globally accounting for 2.3 million new cases and 685,000 deaths in 2020 (1). Ki-67 index is an important marker to indicate tumor cell proliferation and an independent predictive and prognostic factor for early-stage BC patients (2). A high expression level of Ki-67 is associated with a higher recurrence and a poorer patient survival (3, 4), generally indicating more active cell division and proliferation, as well as more aggressiveness, while accompanying by a better efficacy of anti-proliferative chemotherapy (5). In addition, The American College of Surgeons Oncology Group Z1031 (ACOSOG Z1031) trial (6) has shown that Ki-67 index at 2 weeks of treatment is significantly associated with recurrence-free survival, and it can predict the prognosis of BC patients undergoing neoadjuvant endocrine therapy (NET). The proliferative activity of the Ki-67 index is important in decision-making of adjuvant treatments for early-stage BC patients, especially the pathological response to NET (3, 6, 7). Therefore, an accurate and non-invasive predictor is required to determine the expression level of Ki-67 in clinical practice.

At present, Ki-67 expression level is mainly determined by immunohistochemical methods based on core needle biopsy tissue or gross examination after surgery. Both puncture and surgery are invasive methods, which are time-consuming, cost-intensive, and non-reproducible. In addition, the expression level of the proliferation marker Ki-67 could dynamically change over the course of treatment (8). Therefore, it is currently impossible to routinely monitor Ki-67 expression level in BC patients, especially in patients who require to continuous monitoring for neoadjuvant therapy. Ultrasound is a simple, real-time, and non-invasive diagnostic method. In particular, shear wave elastography (SWE), a new ultrasound-based technology, can qualitatively and quantitatively analyze the elastic properties of breast tissues (9). This enables clinicians to analyze the changes in the stiffness of the lesions during the course of neoadjuvant therapy (7, 10), and can provide more information for multiple and repeated monitoring of the biological activity of the lesions, which has gradually been clinically recognized (10, 11). A previous research (12) has shown that the maximum value of elastic modulus (P=0.000) and maximum size (P=0.004) of the Ki-67 high-expression group showed significantly higher values than those of the Ki-67 low-expression group. However, the study did not further validate the diagnostic efficacy of the two indicators.

In 2012, Lambin *et al.* (13) firstly proposed the concept of radiomics. Radiomics transforms medical images into collectible, high-fidelity, and high-throughput data, and selected radiomics features are used to develop predictive models and to support clinical decision-making (14, 15). Radiomics provides a stable and non-invasive approach to reflect the heterogeneity of lesions by extracting a large number of imaging features from the ROI through automatic algorithms, and the critical data are proceeded by diversified statistical analysis and data mining methods (16, 17). Radiomics facilitates the quantitative assessment of tumor heterogeneity to a certain extent, and it has shown great advantages in clinical application.

However, previous studies on the prediction of Ki-67 expression level in BC tissues have mainly concentrated on digital mammography (DM) and magnetic resonance imaging (MRI) (18, 19), and the AUC of the validation set was 0.685 and 0.740, respectively, which was limited. A DM scan uses X-rays, whereas an MRI scan uses strong magnetic fields and radio waves. DM scans are more common and less expensive, but MRI scans produce more detailed images. However, none of them can be used as an effective evaluation method. Comparably, ultrasound (US), especially US examination of thyroid and breast, possesses the advantages of universal typeability, non-radiation, high reproducibility, and high discriminatory, which is widely used in clinical practice. Several scholars have extended the application of radiomics to the field of US imaging, concentrated on the identification of benign and malignant breast tumors (20, 21), attempted to predict biological behavior of invasive ductal carcinoma (IDC) (22), performed the differential diagnosis of triple-negative BC and fibroadenoma (23), and predicted the metastatic status of axillary lymph nodes (ALNs) in early-stage IDC patients (24).

To date, few ultrasound-based radiomics studies have predicted Ki-67 expression level in BC patients. The present research aimed to investigate the quantitative radiomic imaging features extracted from US and to establish a radiomics nomogram *via* combination of radiomics features, gray-scale US images, and SWE to noninvasively predict Ki-67 expression level in BC patients.

## Materials and methods

### Patients

**Figure 1:**
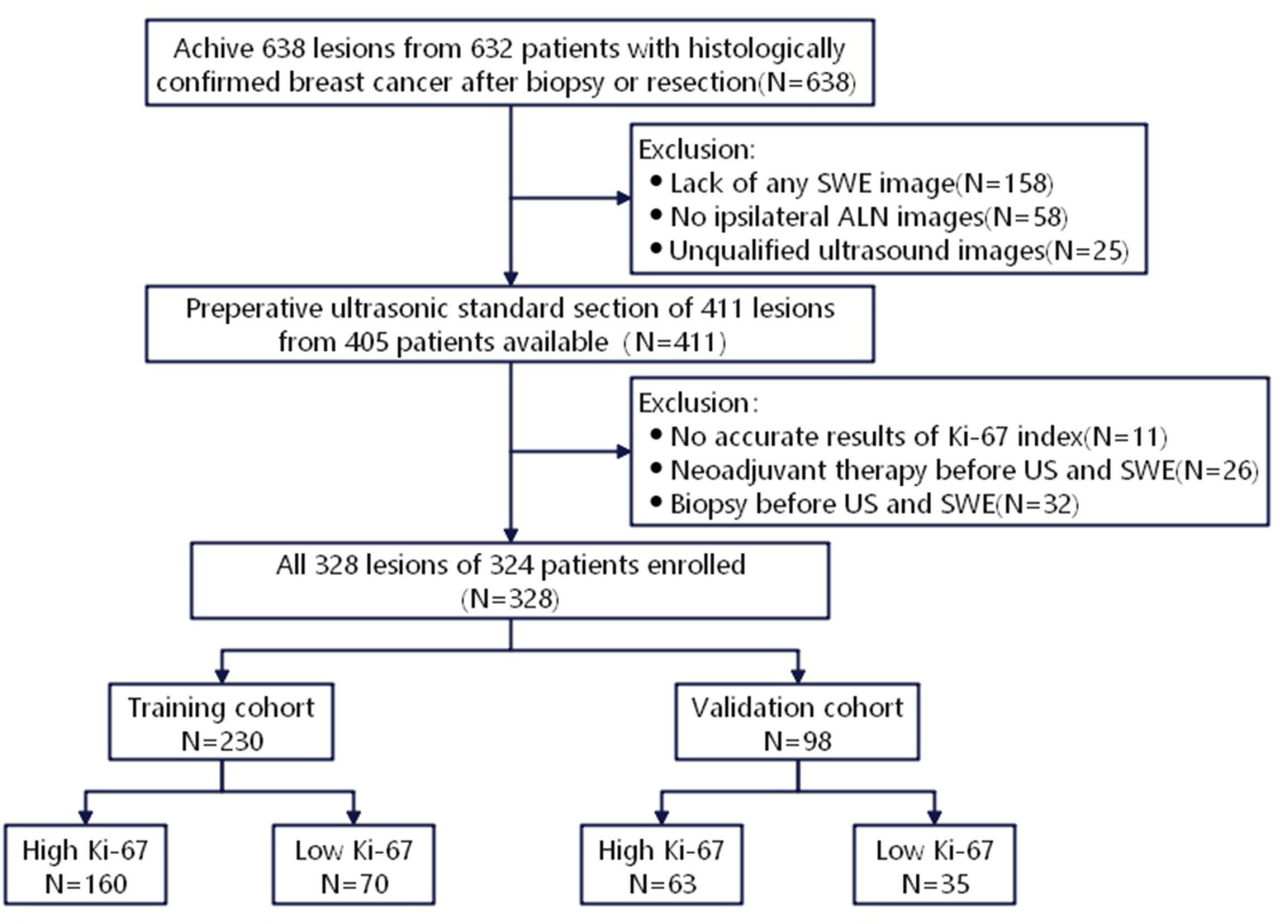
Recruitment pathway for BC patients selection.

This study was approved by the Ethics Committee of the First Affiliated Hospital of Soochow University (Suzhou, China), and the requirement of written informed consent was waived. Patients with BC were diagnosed in our hospital by needle biopsy or surgical pathology from June 2019 to October 2020. Inclusion criteria were as follows: (1) Patients who underwent gray-scale US and SWE; (2) US was completed within two weeks before surgery, followed by needle biopsy and surgical treatment. The exclusion criteria were as follows: (1) Patients who received neoadjuvant therapy; (2) Patients who underwent biopsy before US; (3) Unavailability of data related to immunohistochemical examination or Ki-67 proliferation index. Finally, 328 lesions (324 patients) were involved, including 1 male, 323 females, and 4 bilateral BC patients, who aged 26-88 (average age, 53.03 ± 12.55) years old. All lesions were randomly divided into training cohort (n=230) and validation cohort (n=98) with a ratio of 7:3 (**Figure 1**).

### Clinical data

Baseline clinical and histopathological data, including age, menopause, number of malignant lesions, histological tumor type, and Ki-67 status were retrieved from medical records. Ki-67 score was recorded as the percentage of positively stained malignant cells. According to the St. Gallen International Expert Consensus (25), the criteria for determining the Ki-67 proliferation index are as follows: if Ki-67 ≥ 14%, it is marked as high expression level; otherwise, it is marked as low expression level.

### US data acquisition

US examinations were performed using Resona R7 and Resona R9 ultrasound machines (Mindray Medical International Co., Ltd., Shenzhen, China) with a L14-6 high-frequency linear array probe. The largest section of the ultrasound image was selected to measure the value of the maximum diameter, and the size of the lesion was indicated (< 2 cm, ≥ 2 cm). The lesions were classified according to the American College of Radiology Breast Imaging Reporting and Data System (BI-RADS) classification system (26). Then, we switched to the SWE and attempted to indicate whether lesions in the SWE mode have a stiff rim sign. The stiff rim sign was previously defined as follows (11): compared with the interior of the lesion, the marginal zone of the lesion is red or orange, annular or semi-annular, representing an increase in stiffness; if it was not shown, downward the range and attempt to indicate whether there is a stiff rim sign during the process until the surrounding tissue became orange or red; if a hard ring sign appeared, record the data.

We also evaluated the status of ALN on the ipsilateral side of breast lesions. ALN status based on US findings was assessed according to the following criteria (27): (I) normal ALN was presented with an oval or reniform shape, with a thin (thickness, < 3 mm), even, smooth, C-shaped hypoechoic cortex, and a hyperechoic central fatty hilum; (II) ALN for suspicious metastasis manifested with round shape, focal or eccentric thickened cortex (thickness, > 3 mm), peripheral vascularization, and an indented or effaced fatty hilum. The images were read by 2 radiologists (YZ was reader 1 with 10 years of experience in breast imaging, and MH was reader 2 with 6 years of experience in breast imaging). When there was a discrepancy between two radiologists’ interpretation, the third radiologist (FD, with more than 15 years of experience in breast imaging) made the final decision. All three radiologists were blinded to patients’ clinical and pathological data.

### Radiomics analysis

#### Region of interest (ROI) segmentation

All images were saved in Digital Imaging and Communications in Medicine (DICOM) format. The ROI of BC was drawn manually on the grayscale US image of the maximum cross-section. The segmentation of lesions was conducted by a radiologist with 6 years of experience (reader 2) who was blinded to patients’ Ki-67 status using an open-source imaging platform ITK-SNAP (ver. 3.8.0; http://www.itksnap.org/).

#### Extraction of radiomics features

We extracted 1218 radiomics features, including 9 shape-based features, 234 first-order statistics, and 975 second-order features from each ROI using open-source Pyradiomics packages (ver. 2.12; https://pyradiomics.readthedocs.io/en/2.1.2/) (28). All radiomics features were in accordance with definitions of Imaging Biomarker Standardization Initiative (IBSI) (29). To examine the feature stability, 70 lesions were randomly selected, and the ROI was delineated by another radiologist with 10 years of experience in breast US (reader 1) to assess the consistency between readers. In addition, to assess intra-observer reliability, reader 2 performed the second delineation of ROIs from 70 randomly selected images after 1 week according to the same procedure. The inter- and intra-observer reproducibility of the two radiologists in ROI delineation were measured by the intraclass correlation coefficients (ICCs).

#### Selection of radiomics features

First, ICCs were used to evaluate the interobserver agreement of feature extraction, and features with a good agreement, that is, with ICCs > 0.75, were recruited for further analysis. Second, all of the selected features were normalized with z-score normalization in the training and validation cohorts to achieve a zero mean and unit variance to prevent features in greater numeric ranges from dominating those in smaller numeric ranges. Third, the least absolute shrinkage and selection operator (LASSO) was applied to select the key radiomics features with nonzero coefficients, and a 5-fold cross-validation was conducted to determine an optimal regulation weight (λ). The selected features were used to construct a radiomics signature, and a radiomics score (Rad-score) for each patient was then calculated using a linear combination of the key features weighted by their LASSO coefficients. We finally calculated the area under the receiver operating characteristic (ROC) curve (AUC) value to assess the predictive performance of the Rad-score.

### Development of the radiomics nomogram

Using data from the training cohort, both univariate and multivariate logistic regression analyses were performed to analyze independent predictive factors related to the evaluation of Ki-67 expression level in BC lesions, including clinicopathological characteristics (age, menopausal status, histological type, tumor diameter, stiff rim sign, BI-RADS category, and US-LN status). After performing the multivariate logistic regression analysis, variables with P<0.05 were considered as independent predictive factors and a test of collinearity was performed between the factors and Rad-score. A radiomics nomogram was developed by the multivariate logistic regression analysis. The radiomics signature and the clinicopathological model were also developed in the training cohort to estimate the value of radiomics.

### Validation of the radiomics nomogram

The AUC values were used to evaluate the predictive performance of the Radiomics nomogram in the training and validation cohorts. The DeLong test was used for comparison of the clinicopathological model, radiomics signature, and the radiomics nomogram based on the AUC values. The calibration curves were used to evaluate the agreement between the observed and predicted results by 1000 bootstrap resamples. The clinical applicability of the radiomics nomogram was evaluated through quantifying the net benefit under different threshold probabilities in the validation cohort by decision curve analysis (DCA).

### Statistical analysis

Statistical analysis was conducted with R software (version 4.0.4; http://www.R-project.org). The Student’s t-test or the Mann–Whitney U test was used for comparing differences in continuous variables. The Pearson’s Chi-square test or the Fisher’s exact test was used to compare differences in categorical variables. The *glm* function was used in the univariate and multivariate logistic regression analyses. The LASSO regression analysis was performed using the “caret” and “glmnet” packages. The “pROC” package was utilized to plot the ROC curves. AUC values were used to estimate the performance of the models, and were compared using the DeLong test. Nomogram construction was carried out with the “rms” package. The “*calibration curve*” function was used to plot the calibration curves. The DCA was conducted by the “rmda” package. A two-sided P < 0.05 was considered statistically significant.

## Results

### Clinicopathological characteristics

The clinicopathological characteristics in the training and validation cohorts are shown in **Table 1**. The univariate analysis showed that parameters, such as age, menopausal status, number of patients with high Ki-67 expression level, US-reported maximum tumor diameter (MTD), histopathological type, stiff rim sign, and ultrasound BI-RADS classification were not significantly different between the training and validation cohorts (all P>0.05), except for US-reported ALN status (P=0.027).

**Table 1:** Comparison of descriptive characteristics of the training and validation cohorts.

Characteristic	Training cohort (n=230)	Validation cohort (n=98)	*P*value
**Age (years)**	52.61 ± 12.41	53.90 ± 12.97	0.396
**High Ki-67 expression**	160 (69.6%)	63 (64.3%)	0.348
**Menopausal status**			
Premenopausal	96 (41.7%)	44 (44.9%)	0.597
Postmenopausal	134 (58.3%)	54 (55.1%)	
**US-reported MTD**			
<2cm	99 (43.0%)	53 (54.1%)	0.067
≥2cm	131 (57.0%)	45 (45.9%)	
**Histological type**			
Ductal carcinoma in situ	23 (10.0%)	11 (11.2%)	0.422
Invasive ductal carcinoma	189 (82.2%)	76 (77.6%)	
Invasive lobular carcinoma	5 (2.2%)	1 (1.0%)	
Others	13 (5.7%)	10 (10.2%)	
**Stiff rim sign**			
No	75 (32.6%)	34 (34.7%)	0.714
Yes	155 (67.4%)	64 (65.3%)	
**BI-RADS category**			
3	1 (0.4%)	2 (2.0%)	0.279
4A	56 (24.3%)	28 (28.6%)	
4B	76 (33.0%)	34 (34.7%)	
4C	62 (27.0%)	26 (26.5%)	
5	35 (15.3%)	8 (8.2%)	
**US-reported LN status**			
LN-negative	150 (65.3%)	76 (77.6%)	0.027
LN-positive	80 (34.7%)	22 (22.4%)	

MTD, maximum tumor diameter; LN, lymph node.

The univariate analysis showed that US-reported MTD, histopathological type, stiff rim sign, ultrasound BI-RADS classification, and US-reported ALN status were correlated with Ki-67 expression level in the training cohort (**Table 2**). Then, these characteristics were imported to the multivariate logistic regression analysis. In the multivariate logistic regression analysis, US-reported MTD, stiff rim sign, and US-reported ALN status (all P<0.05) were proven to be the independent predictive factors in identifying high and low expression levels of Ki-67 (**Table 3**). There was no multicollinearity problem between these three factors and Rad-score. The clinicopathological model constructed by the three factors performed well, with the AUC values of 0.883 (95% CI: 0.835-0.931) and 0.844 (95% CI: 0.758-0.931) in the training and validation cohorts, respectively.

**Table 2:** Characteristics associated with Ki-67 expression status in training and validation cohorts.

	Training cohort(n=230)			Validation cohort(n=98)		
	Low expression	High expression	*P* value	Low expression	High expression	*P* value
**No. of Lesions**	70	160		35	63	
**Age (years)**	54.71 ± 12.03	51.69 ± 12.50	0.089	54.06 ± 15.72	53.81 ± 11.29	0.935
**Menopausal status**						
Premenopausal	28 (40.0%)	68 (42.5%)	0.724	16 (45.7%)	28 (44.4%)	0.904
Postmenopausal	42 (60.0%)	92 (57.5%)		19 (54.3%)	35 (55.6%)	
**US-reported MTD**						
<2cm	50 (71.4%)	49 (30.6%)	<0.001	28 (80.0%)	25 (39.7%)	<0.001
≥2cm	20 (28.6%)	111 (69.4%)		7 (20.0%)	38 (60.3%)	
**Histological type**						
Ductal carcinoma in situ	17 (24.3%)	6 (3.8%)	<0.001	6 (17.1%)	5 (7.9%)	0.031
Invasive ductal carcinoma	48 (68.6%)	141 (88.1%)		22 (62.9%)	54 (85.7%)	
Invasive lobular carcinoma	1 (1.4%)	4 (2.5%)		0 (0.0%)	1 (1.6%)	
Others	4 (5.7%)	9 (5.6%)		7 (20.0%)	3 (4.8%)	
**Stiff rim sign**						
No	52 (74.3%)	23 (14.4%)	<0.001	26 (74.3%)	8 (12.7%)	<0.001
Yes	18 (25.7%)	137 (85.6%)		9 (25.7%)	55 (87.3%)	
**BI-RADS category**						
3	1 (1.4%)	0 (0.0%)	<0.001	0 (0.0%)	2 (3.2%)	0.120
4A	28 (40.0%)	28 (17.5%)		15 (42.8%)	13 (20.6%)	
4B	25 (35.7%)	51 (31.9%)		11 (31.4%)	23 (36.5%)	
4C	14 (20.0%)	48 (30.0%)		8 (22.9%)	18 (28.6%)	
5	2 (2.9%)	33 (20.6%)		1 (2.9%)	7 (11.1%)	
**US-reported LN status**						
LN-negative	64 (91.4%)	86 (53.7%)	<0.001	33 (94.3%)	43 (68.3%)	0.003
LN-positive	6 (8.6%)	74 (46.3%)		2 (5.7%)	20 (31.7%)	

MTD, maximum tumor diameter; LN, lymph node.

**Table 3:** Multivariate analysis of clinic-radiological characteristics in training cohort.

Characteristic	P value	β	OR value	95%CI
US-reported MTD	0.005	1.097	8.015	1.402~6.404
Stiff rim sign	<0.001	2.644	46.302	6.568~30.116
US-reported LN status	<0.001	1.849	13.100	2.334~17.287
constant	<0.001	-1.665	23.748	

MTD, maximum tumor diameter; LN, lymph node.

### Radiomics analysis

**Figure 2:**
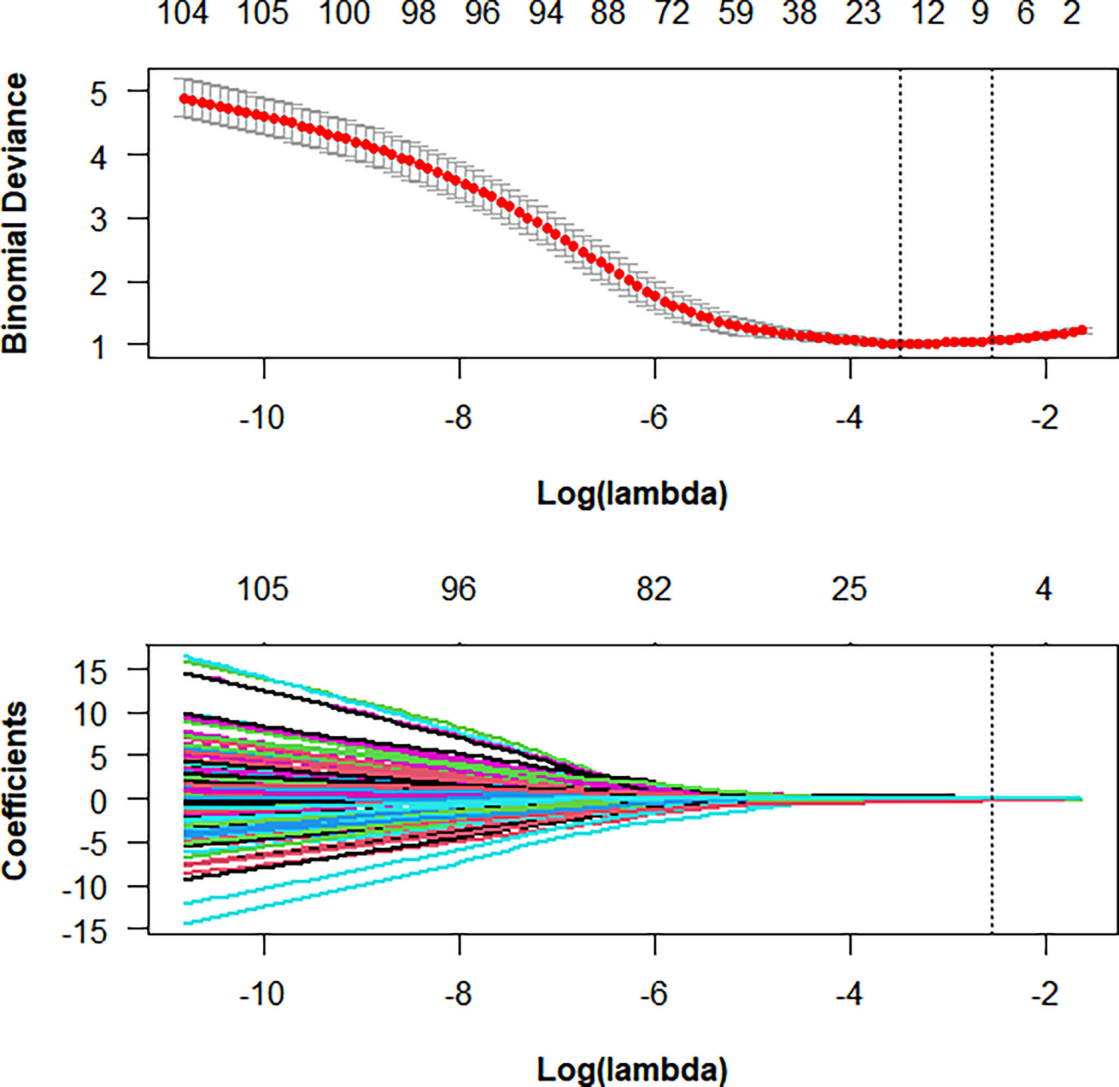
Radiomics feature selection using LASSO logistic regression in the training cohort.

**Figure 3:**
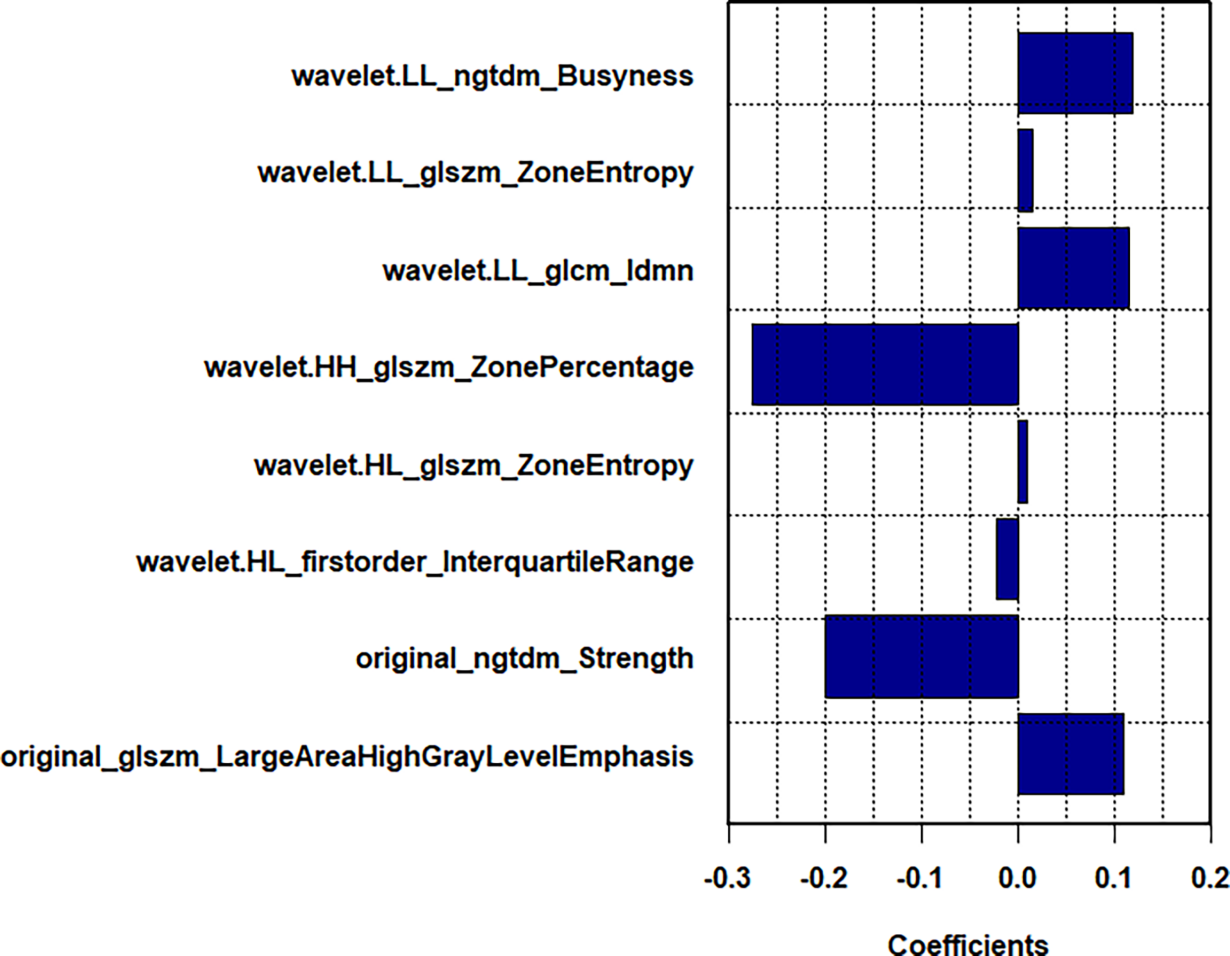
In LASSO regression, a coefficient profile plot was drawn and resulted in 8 radiomic features with nonzero coefficients.

Among 1218 radiological features extracted, 985 features with ICC greater than 0.75 in both inter-observer and intra-observer were screened. Then, the optimal feature data set with the least cross-validation binomial deviance was selected by the LASSO method, and non-zero coefficients were defined as the weight for each selected feature, which indicated the correlation between the feature and Ki-67 expression level. Finally, 8 relevant features, including 1 first-order feature and 7 second-order features were selected (**Figures 2**, **3**). The Rad-score of each lesion was calculated by the 8 selected features. In the training cohort, Rad-score showed a significant difference between high and low expression levels of Ki-67 in breast lesions (P<0.001). The AUC values of the radiomics signature were 0.821 (95% CI: 0.764-0.880) and 0.713 (95% CI: 0.612-0.814) in the training and validation cohorts, respectively.

### Development of the radiomics nomogram

**Figure 4:**
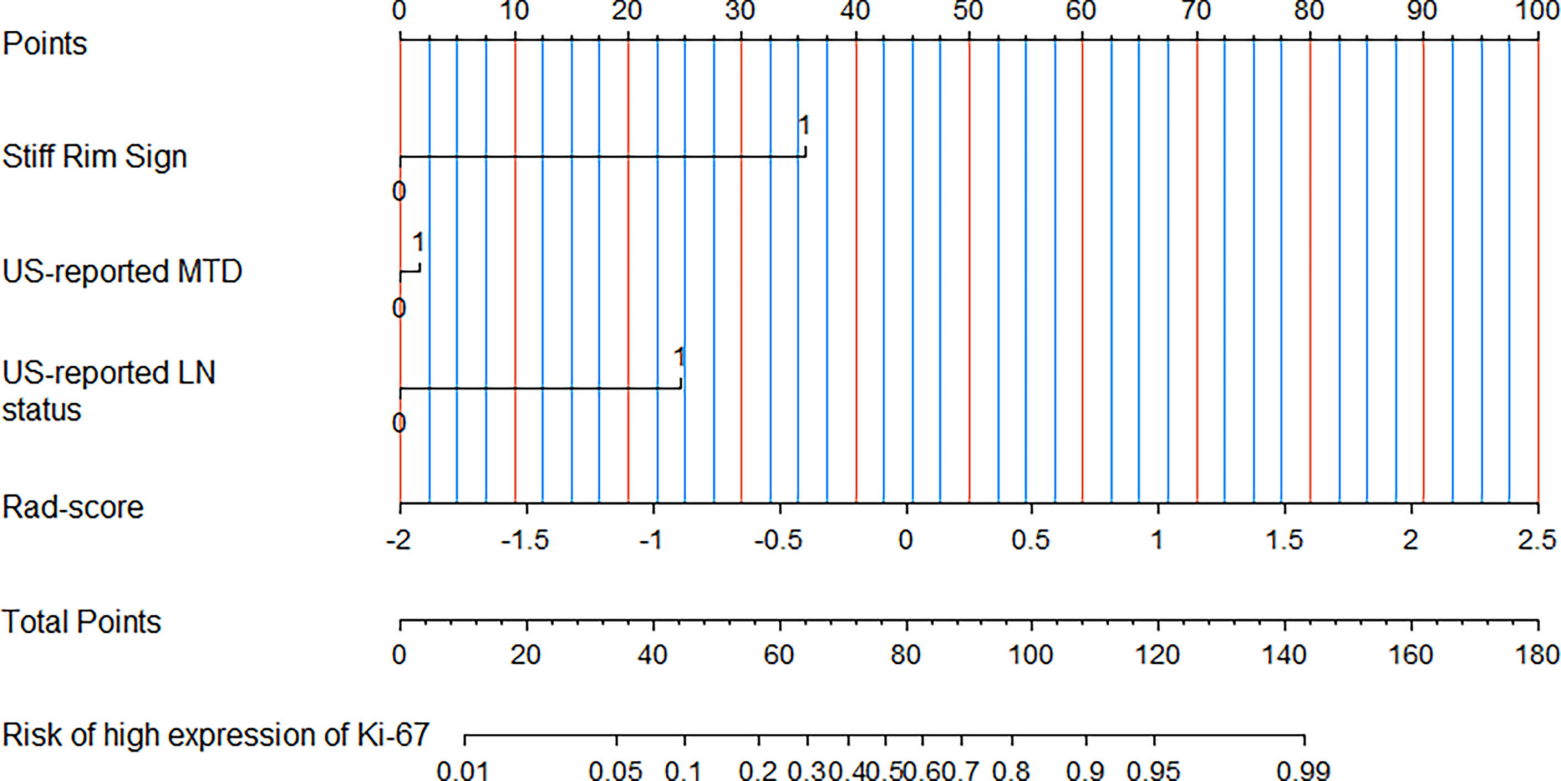
A radiomics nomogram was developed with stiff rim sign, US-reported MTD, US-reported LN status and Rad-score for the prediction of Ki-67 expression status in the training cohort.

In the multivariate logistic regression analysis, US-reported MTD, stiff rim sign, US-reported ALN status, and Rad-score (all P<0.05) were found to be the independent predictive factors in identifying high and low expression levels of Ki-67. The radiomics nomogram was developed with the 4 efficient features, which showed the best performance in the three models with AUC values of 0.904 (95% CI:0.860-0.948) and 0.890 (95% CI: 0.817-0.964) in the training and validation cohorts, respectively (**Figure 4**). The DeLong test showed that there was a significant difference between the radiomics nomogram and the clinicopathological model in the training cohort (P=0.032), while showed no significant difference in the validation cohort (P=0.139). There was a significant difference between the radiomics nomogram and the radiomics signature in the training cohort (P=0.001) and validation cohort (P=0.000), respectively (**Table 4**). In the training and validation cohorts, the sensitivity of the radiomics nomogram reached 86.9% and 98.4%, respectively. The specificities were 85.7% (training cohort) and 74.3% (validation cohort), respectively.

**Table 4:** AUC comparison of three prediction models.

Model	Training cohort		Validation cohort	
	Z value	P value	Z value	P value
Clinic-radiological model VS radiomics signature	2.064	0.039	2.293	0.022
Clinic-radiological model VS Radiomics nomogram	-2.139	0.032	1.48	0.139
Radiomics signature VS Radiomics nomogram	-3.487	0.0005	3.627	<0.001

### Validation of the nomogram

**Figure 5:**
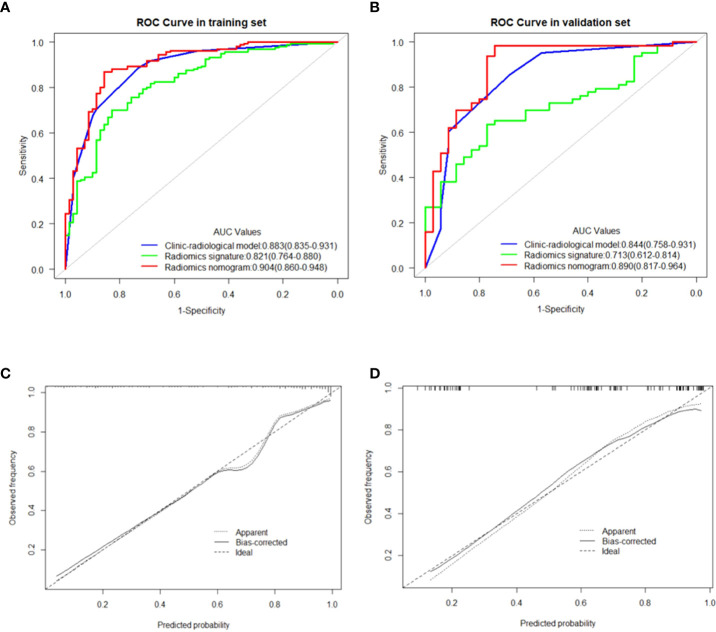
**(A, B)** showed the comparison of receiver operating characteristic curves between the clinic-radiological model, radiomics signature and radiomics nomogram in the training and validation cohorts, respectively. Calibration curves of the radiomics nomogram in the training cohort **(C)** and validation cohort **(D)**. The 45 straight line represents a perfect match between the actual (Y-axis) and nomogram-predicted probabilities (X-axis), and the dotted lines represent the predictive performance of the nomogram.

**Figure 6:**
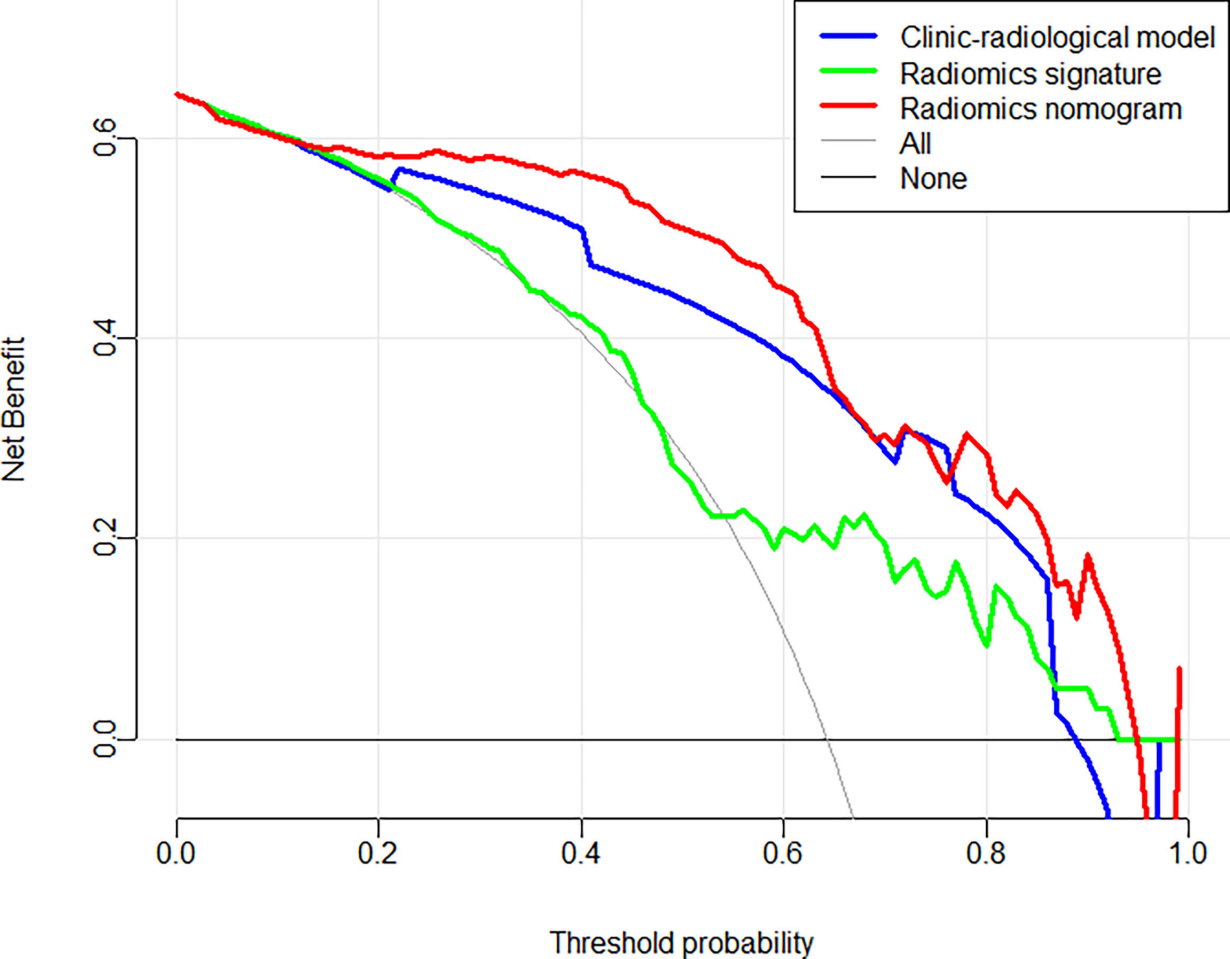
Decision curve analysis for each model in predicting Ki-67 expression status for BC patients.

In the training and validation cohorts, the calibration curves of the radiomics nomogram demonstrated that the bias curves were both close to the ideal line in the figures and showed a good consistency between predicted and actual outcomes (**Figure 5**). The Hosmer-Lemeshow test also yielded a non-significant P value of 0.62 in the training cohort and 0.41 in the validation cohort, suggesting no deviation from the good fit. **Figure 6** summarizes the clinical application of prediction models by DCA. Compared with clinical-radiological model or radiomics signature, the radiomics nomograms improved the prediction of Ki-67 expression level and provided more net benefits at a wide range of risk thresholds.

## Discussion

In the present study, a radiomics signature was constructed to predict the Ki-67 expression level using a machine learning technique based on US images, and it was proved to be an independent predictor. More importantly, our research indicated that the radiomics nomogram combined with the radiomics signature and clinicopathological variables (including SWE) could preoperatively predict the Ki-67 expression level in BC patients with a satisfactory performance.

Radiomics has shown a great capability in differentiating benign and malignant tumors (20, 21, 30), discriminating molecular subtypes (31), distinguishing between benign and malignant non-mass enhancement lesions (32), predicting ALN metastasis (24, 33), and responding to neoadjuvant chemotherapy (34) based on MRI, CT or US findings. A recent research showed that MRI- or digital breast tomosynthesis-based radiomics can be used for prediction of Ki-67 expression level in BC patients. Zhang *et al.* (35) used 11 radiomics features extracted from apparent diffusion coefficient (ADC) maps of sequences on MRI to construct a radiomics model to predict Ki-67 expression level, with the AUC of 0.75 in training dataset and 0.72 in test dataset. Tagliafico *et al.* (36) combined the most 5 predictive features extracted from digital breast tomosynthesis, and achieved the best AUC of 0.698. However, there are few studies on the prediction of Ki-67 expression level by ultrasound-based radiomics. Compared with earlier studies (35, 36), this study, on the one hand, adopted a more convenient and economical ultrasound imaging means, while also taking into account the hardness assessment of shear wave elastography; on the other hand, the training and validation sets achieved AUC values of 0.904 and 0.890, with better diagnostic performance.

In the present study, radiomics was conducted to analyze the US images of breast tumors, and 8 features were extracted from each primary lesion image. Among the radiomics features, wavelet.HH_glszm_ZonePercentage showed the strongest correlation with the Ki-67 expression level, followed by original_ngtdm_Strength. These selected radiomics features, excluding first-order_Interquartile_Range, are higher-order features and represent the heterogeneity of the tumor and the slight difference of the gray and textural features (37).

Regarding the Zone Percentage, a higher value indicates that a larger portion of the ROI consists of small zones, demonstrating that the texture is finer. The results of the present study showed that the Zone Percentage value is negatively correlated with the Ki-67 expression level, indicating that BC lesions with a high Ki-67 expression level have a lower zone percentage value and a rougher textural distribution. Idmn gray-level co-occurrence matrix (GLCM) feature is one of the parameters of GLCM, which has been widely used in textural analysis (20), and it could describe the homogeneous echo pattern. According to results of the present research, a high Ki-67 expression level correlated with Idmn GLCM feature may accompany by inhomogeneity. In the cellular level, the high Ki-67 expression level may lead to the high proliferative activity of tumor cells, and the tumor area is prone to ischemia, hypoxia, and even liquefaction or necrosis, resulting in inhomogeneity within the tumor. A previous study (38) revealed that entropy was closely associated with tumor invasiveness. In the present study, “zone entropy”, measuring the uncertainty and randomness in the distribution of zone sizes and gray levels, was the important feature in high-order textural features and was positively correlated with a high Ki-67 expression level.

We, in the current study, developed a new radiomics nomogram that incorporated radiomics signature with US-reported tumor size, stiff rim sign, and US-reported ALN status to further improve predictive accuracy for Ki-67 expression level in BC patients. These four parameters were obtained non-invasively and did not need puncture biopsy, surgery or immunohistochemical analysis. Therefore, similar to Yu *et al.*’s research (22) who predicted metastatic status of ALNs, the greatest advantage of our nomogram was that Ki-67 expression level could be assessed non-invasively before surgery. Meanwhile, compared with the clinicopathological model or radiomics signature used alone, the diagnostic efficiency of the proposed radiomics nomogram was improved up to 0.904 (training cohort) and 0.895 (validation cohort) of AUC. Moreover, in this study, although the prediction accuracy of Rad-score was slightly lower than that of the clinicopathological model, the radiomics nomogram combined with Rad-score was accompanied by a higher accuracy than the clinicopathological model or Rad-score only. This showed that radiomics could be used as an important supplement to clinicopathological data to identify high and low expression levels of Ki-67 in BC lesions. Using calibration curves, the results of our research revealed that the predictive probability had a high agreement with the actual probability. DCA showed that US-based radiomics nomogram provided more net benefit for a greater number of BC patients compared with the clinicopathological model or radiomics signature, indicating that Rad-score increases the clinical value and reduces US-dependent risk factors for clinical decision-making.

Several advantages of the present study should be pointed out. First, we, for the first time, presented the use of US-based radiomics to predict Ki-67 expression level, and verified its effectiveness and stability. Second, in addition to the Rad-score, the final prediction model incorporated clinicopathological features and elastic properties of breast tissues from SWE to comprehensively evaluate the heterogeneity of BC lesions. These data were non-invasive and reproducible, and were not obtained by puncture biopsy, surgery or immunohistochemical analysis. Third, we constructed a radiomics nomogram, which showed a great potential in predicting disease progression and prognosis (39). The application of the proposed radiomics nomogram could assist clinicians to select the most appropriate treatment plan based on the predicted probability.

Our study has also several limitations. First, due to the limitation of ultrasonic scanning probe, the radiomics features were all extracted from two-dimensional (2D) images. Compared with three dimensional (3D) features, 2D features may lack some information that may fully describe the features of the entire lesion. However, a previous study showed that 2D features extracted from MR patterns performed better than 3D features in lung cancer (40). Further investigations should be conducted to explore the potential significance of radiomics of 3D ultrasound for predicting the Ki-67 expression level in BC patients. Second, all US data were acquired from the same US machine, and the prediction model was not validated by other US machines. Therefore, the clinical applicability of the proposed predictive model needs to further evaluation and validation. Third, in this retrospective study, the establishment and validation of the combined model for prediction of the Ki-67 expression level were carried out in a single institution with the limited sample size, thus, a larger standardized sample size and an external validation by multicenter studies will be essential.

In conclusion, a novel radiomics nomogram that combined the clinicopathological characteristics and US-based radiomics signature demonstrated promising predictive performance and clinical applicability for predicting Ki-67 expression level in BC patients. The US-based radiomics has the potential to be used as a non-invasive approach to develop the treatment strategies and to assist clinicians in making reliable clinical decisions.

## Data availability statement

The original contributions presented in the study are included in the article/supplementary material. Further inquiries can be directed to the corresponding authors.

## Ethics statement

The studies involving human participants were reviewed and approved by the Ethics Committee of the First Affiliated Hospital of Soochow University. Individual consent for this retrospective analysis was waived.

## Author contributions

JL, XW, and MH contributed equally to this study. Conception and design of the study: FD and YD. Ultrasound and SWE data acquisition: MH, YZ, and FD. Clinical and pathological data collection: JL, LZ, and WW. Analysis and interpretation of data: XW, ZZ, JH, and JL. Drafting the manuscript: JL. Discussion and manuscript revision: XW, MH, ZZ, YD, and FD. All authors contributed to the article and approved the submitted version.

## Funding

This study was supported by the Suzhou Clinical Key Diseases Diagnosis and Treatment Technology Special Project (LCZX202104).

## Conflict of interest

The authors declare that the research was conducted in the absence of any commercial or financial relationships that could be construed as a potential conflict of interest.

## Publisher’s note

All claims expressed in this article are solely those of the authors and do not necessarily represent those of their affiliated organizations, or those of the publisher, the editors and the reviewers. Any product that may be evaluated in this article, or claim that may be made by its manufacturer, is not guaranteed or endorsed by the publisher.
